# High burden of anemia among pregnant women in Tanzania: a call to address its determinants

**DOI:** 10.1186/s12937-021-00726-0

**Published:** 2021-07-08

**Authors:** Bruno F. Sunguya, Yue Ge, Linda Mlunde, Rose Mpembeni, Germana Leyna, Jiayan Huang

**Affiliations:** 1 School of Public Health and Social Sciences, Muhimbili University of Health and Allied Sciences, Dar es Salaam, Tanzania; 2 Global Health Institute, School of Public Health, Fudan University, Shanghai, China; 3 Key Laboratory of Health Technology Assessment, National Health Commission, Shanghai, China; 4 Implementation Science Tanzania, Dar es Salaam, Tanzania; 5 Tanzania Food and Nutrition Center, Dar es Salaam, Tanzania

**Keywords:** Anemia, Pregnancy, Demographic and health survey, Tanzania, Women of reproductive age

## Abstract

**Background:**

Anemia in pregnancy is behind a significant burden of maternal mortality and poor birth outcomes globally. Efforts to address it need evidence on trends and its pertinent factors as they vary from one area to another.

**Methods:**

We pooled data of 23,203 women of reproductive age whose hemoglobin levels were measured from two Tanzania Demographic and Health Surveys (TDHS). Of them, 2,194 women were pregnant. Analyses employed descriptive analyses to determine the burden of anemia, its characteristics, and severity; GIS mapping to determine the regional changes of anemia between 2005 and 2015; and logistic regression to determine the remaining determinants of anemia among pregnant women using Stata 15.

**Results:**

The burden of anemia among pregnant women in Tanzania has remained unprecedently high, and varies between regions. There was no significant decline of anemia in general between the two periods after adjusting for individual, households, reproductive, and child characteristics [AOR = 0.964, 95% CI = 0.774–1.202, *p* = 0.747). Anemia is currently prevalent in 57% of pregnant women in Tanzania. The prevalence is more likely to be higher among women aged 15–19 years than those aged between 20–34 years. It is more likely to be prevalent among those within large families, with no formal education, food insecurity, lack of health insurance, had no antimalaria during pregnancy, and had low frequency of ANC attendance. On the other hand, delivery in a health facility may be potentially protective against anemia.

**Conclusions:**

Anemia in pregnancy remained persistently high and prevalent among 57% of pregnant women in Tanzania. Efforts to address anemia are crucial and need to be focused in regions with increasing burden of anemia among pregnant women. It is imperative to address important risk factors such as food insecurity, strengthening universal health coverage, empowering women of reproductive age with education and especially nutritional knowledge and advocating for early antenatal booking, attendance, and facility delivery.

**Supplementary Information:**

The online version contains supplementary material available at 10.1186/s12937-021-00726-0.

## Background

Globally, about one in three women of reproductive age (WRA) is estimated to suffer from anemia, presenting with haemoglobin (Hb) below 11 g/dl [1]. The burden is even higher among pregnant women. The 2011 Nutrition Impact Model Study projected anemia to be prevalent among 38% of pregnant women globally [2]. Like other forms of chronic malnutrition, such unprecedented burden remains higher in low- and middle-income countries compared to developed nations. Within the Sub-Saharan Africa, anemia among pregnant women is the highest in West Africa, with the prevalence of 56% [1]. Anemia during pregnancy is associated with a number of adverse events for both the mother and her offspring [3, 4]. Chiefly, it has been an important cause of maternal and perinatal mortality globally [5, 6]. Severity of the adverse outcomes of anemia in pregnancy depends largely on severity of anemia itself [6]. Some of the outcomes include premature labor and rupture of membranes [7], pregnancy induced hypertensive diseases including eclampsia and pre-eclampsia [8, 9], risk of caesarean sections, and poor child outcomes including low birth weight, small for gestation age, still birth, and neonatal deaths [7]. Anemia in pregnancy may also predispose a child to early developmental challenges [10] and subsequent early burden of undernutrition [11].

Like in other low and middle-income countries, anemia remains one of the important indirect causes of 14.5% of maternal deaths in Tanzania [12]. Despite efforts in place to address persistent maternal deaths in the country, results have not been promising, calling for further efforts in mitigating the important determinants such as anemia [13]. Currently, maternal mortality rate stands at 576 deaths per 100,000 live births [13]. This burden can be attributed by a number of direct and indirect causes including unprecedented burden of anemia in pre-pregnancy and the pregnancy period [14]. Anemia among pregnant women in Tanzania commonly associates with inadequate dietary diversity coupled with inadequate daily meal intake [15, 16]. Other forms that contribute to such burden include diseases such as malaria, parasitic worms which are also preventable [17].

Interventions, like iron supplementation [18] and deworming [19] to address anemia have resulted into a small but notable decline of prevalence of anemia among vulnerable populations in Tanzania. For example, it has declined among children under the age of 5 years old from 71% in 2005 to 59% in 2015 in Tanzania [20]. Similarly, anemia has significantly declined among women of reproductive age 48% in 2005 to 45% in 2015 [13]. However, lack of similar analyses in Tanzania among pregnant women who carry significant risks needed to be addressed and therefore a rationale of this study.

Like for other sub-populations in Tanzania [20], determinants of anemia among pregnant women may also vary from one region to another, since food productivity varies between regions, and international organizations like the World Bank have different interventions on anemia in different regions [21]. Efforts to address this burden therefore need tailored approach and targeted interventions. Such evidence is not available to affect policy and guidelines in the country. Variation of magnitudes and determinants were found in our previous publications among women and children [20, 22]. In addressing these scientific gaps, this study therefore aimed to first determine the magnitude of anemia among pregnant women in Tanzania and changes of this burden in a period of 10 years. Second, to determine the regional differences and changes over time between regions. Third, to characterize anemia in pregnancy in order to understand the vulnerabilities among pregnant women and to examine the determinants of anemia in pregnancy for policy and implementation recommendations in Tanzania.

## Methods

We conducted a cross-sectional study using secondary data from the nationally representative Tanzania Demographic and Health Surveys (TDHS) of 2004–2005 [23] and the latest survey of 2015–2016 [13]. The authors chose these datasets owing to the availability of anemia data that was introduced to DHS program in 2004–2005 for Tanzania. DHS datasets are generated from cross-sectional studies conducted in all regions. For Tanzania, it includes data from the mainland and Zanzibar islands. The National Bureau of Statistics (NBS) has been the leading institution in the mainland, while Zanzibar Bureau of Statistics (ZBS) has been leading these studies in the isles through the USAID funding and technical leadership of MEASURES (https://dhsprogram.com/). Since 1992, DHS surveys have been vital sources for nationally representative data on maternal, child, and household’s characteristics related to health, wellbeing, health services delivery, and other social determinants of health in Tanzania. Specifically, for nutrition research, data is collected from children and women on their anthropometric measurements and can be used to generate nutrition status variables, biological markers for micronutrients such as iron, vitamin A, and iodine. These variables and biomarkers can help to estimate the prevalence of anemia, vitamin A deficiency, and iodine deficiency in general population and sub-populations such as vulnerable populations like women of reproductive age and pregnant women.

This analysis aimed to examine the prevalence of anemia among pregnant women during the respective surveys. It therefore pooled data of a total of 23,203 women of reproductive age whose hemoglobin levels were measured, and out of them, a sub-population of 2,194 women who were pregnant during the respective surveys were analyzed further. The distribution of this population was as follows: for the 2004–2005 survey, a total of 10,139 WRA were pooled of which 1,075 were pregnant at the time of survey; and in 2015–2016 survey, a total of 13,064 WRA were pooled of which, 1,119 confirmed to be pregnant at the time of the survey.

In both surveys, the main outcome variable was anemia defined as a blood haemoglobin (Hb) level below 11.0 g/dl in pregnant women and below 12.0 g/dl in non-pregnant women [24]. Anaemia among women was measured by collecting and testing capillary blood from a finger prick with the HemoCue 201 + analyzer [25]. An adjustment of the hemoglobin count is made for altitude with the following formulas: adjust = -0.032*alt + 0.022*alt^**2**^, **adjHb = Hb-adjust** if adjust > 0, where **adjust** is the amount of the adjustment, **alt** is altitude in 1,000 feet (converted from meters by dividing by 1,000 and multiplying by 3.3), **adjHb** is the adjusted hemoglobin level, and Hb is the measured hemoglobin level in grams per deciliter. No adjustment is made for altitudes below 1,000 m. Similarly, an adjustment is made for women who smoke (if information was collected): For women who smoke less than 10 cigarettes per day, no adjustment are made; for women who smoke 10–19 cigarettes per day, adjust Hb (g/dl) concentration by –0.3; for women who smoke 20–39 cigarettes per day, adjust Hb (g/dl) concentration by –0.5; for women who smoke 40 or more cigarettes per day, adjust Hb (g/dl) concentration by –0.7; for women who smoke unknown quantity or non-cigarettes smoking, adjust Hb (g/dl) concentration by –0.3 [2, 24]. In pregnant women, mild anemia is defined as hemoglobin count between 10.0 and 10.9 g/dl; moderate anemia is defined as hemoglobin count between 7.0 and 9.9 g/dl; severe anemia is defined as hemoglobin count less than 7.0 g/dl [24]. Independent variables included demographic, household, and other maternal and child characteristics based on previous literatures.

Further within the independent variables, the individual characteristics included age (in years); highest education level attained; current marital status, use of any form of tobacco; and having any form of health insurance. Other variables under households’ characteristics included place of residence whether was in urban or rural, number of household’s members, household’s food security, and weighted wealth index. Like in our previous studies [20, 22], weighted wealth index was computed using principal component analysis and factor analyses of the household assets ownership. The factor loadings which are sample weights are summed to generate the weighted wealth index. DHS data presents the quintiles of such weighted wealth index categories as poorest, poorer, middle, richer, and richest. Reproductive and child characteristics included number of children ever born, age at the first child birth, gestation age at first antenatal booking, number of antenatal clinic visit, provision and use of folic acid and iron tablets during pregnancy, place of delivery, and deworming during the last pregnancy.

Data was analyzed using descriptive statistics, regression analyses, and using GIS mapping to indicate the changes within regions as far as anemia in pregnancy is concerned. For descriptive analyses, Pearson’s Chi-square test was used to examine the differences of anemia with regards demographic, household, and other health-related characteristics that are hypothesized to be related to anemia among pregnant women. Further, the regional differences in the burden of anemia among pregnant women were examined over the period of 10 years and color-coded to show the magnitude of change between the regions.

To examine the independent change of anemia prevalence among pregnant women in Tanzania, the 2004–2005 and 2015–2016 datasets were analyzed using multivariable logistic regression analysis. In this regression, we studied the association between anemia among pregnant women and survey year, with anemia as the dependent variable and survey year (treated as a categorical variable), as well as demographic, households, and health-related characteristics as the independent variables. Another multivariable logistic regression analysis was conducted to determine the remaining determinants of anemia among pregnant women in Tanzania using the 2015–2016 datasets, with anemia as the dependent variable and demographic, households, and health-related characteristics as the independent variables. We applied the sampling weight generated by the TDHS to handle the sampling design. Data were analyzed using Stata version 15 software. Since this was a secondary analysis of DHS data, we did not require a separate ethical approval. The primary data collection had followed and adhered to all ethical considerations including informed consent and permission to use the data was sought through the DHS website (https://dhsprogram.com/).

## Results

### The prevalence of anemia in pregnancy between 2005 and 2015

The prevalence of anemia has remained 57.1% among pregnant women in 2015 from 58.2% in 2005 (*P* = 0.680), which means there has not be any significant decline for over 10 years between the surveys (Table 1). Among pregnant women with anemia, a sheer majority had a moderate form in both surveys. There has been a significant decline of severe form of anemia from 2.7% in 2005 to 1.2% in 2015 (*p* = 0.017).

**Table 1:** The burden of anemia among women of reproductive age stratified by pregnancy status

**Variable**	**2004–2005**		**2015–2016**		***P*-value**
	**N**	**%**	**N**	**%**	
Non-pregnant	4278	47.2	5218	43.7	0.009
Pregnant	625	58.2	639	57.1	0.680
Mild	244	22.7	283	25.3	0.230
Moderate	353	32.8	342	30.6	0.383
Severe	29	2.7	13	1.2	0.017

The *P*-values were taken from Pearson’s Chi-square test

**Fig. 1:**
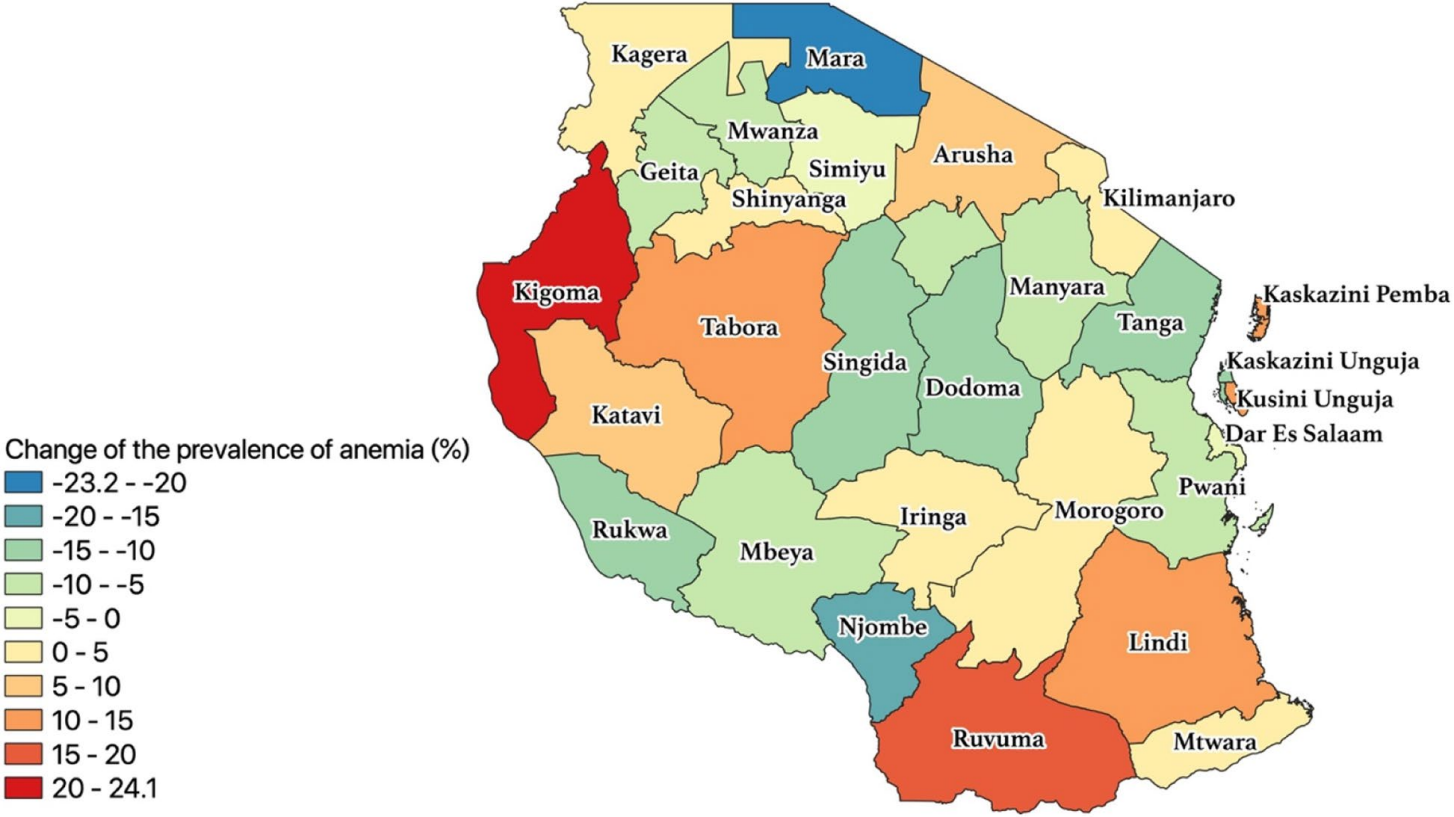
GIS Mapping showing changes in the burden of anemia among pregnant women between 2005 and 2015

Despite the stagnation of the overall burden of anemia in pregnancy, analysis by region showed that the change over time differed between regions (Fig. 1). A total of 15 regions observed a decline of anemia among pregnant women in Tanzania. The magnitude of change was observed more in Mara (23.2%), Njombe (16.5%), Singida (14.7%), Dodoma (12.4%), and Rukwa (11.9%). On the other hand, we noted an increase in the burden of anemia 16 regions and at a various level of change. Regions with a higher increase of anemia included Kigoma (24.1%), Ruvuma (19.7%), Kusini Pemba (12.5%), Kusini Unguja (12.3%), and Lindi (11.6%) (see Additional file 1).

**Fig. 2:**
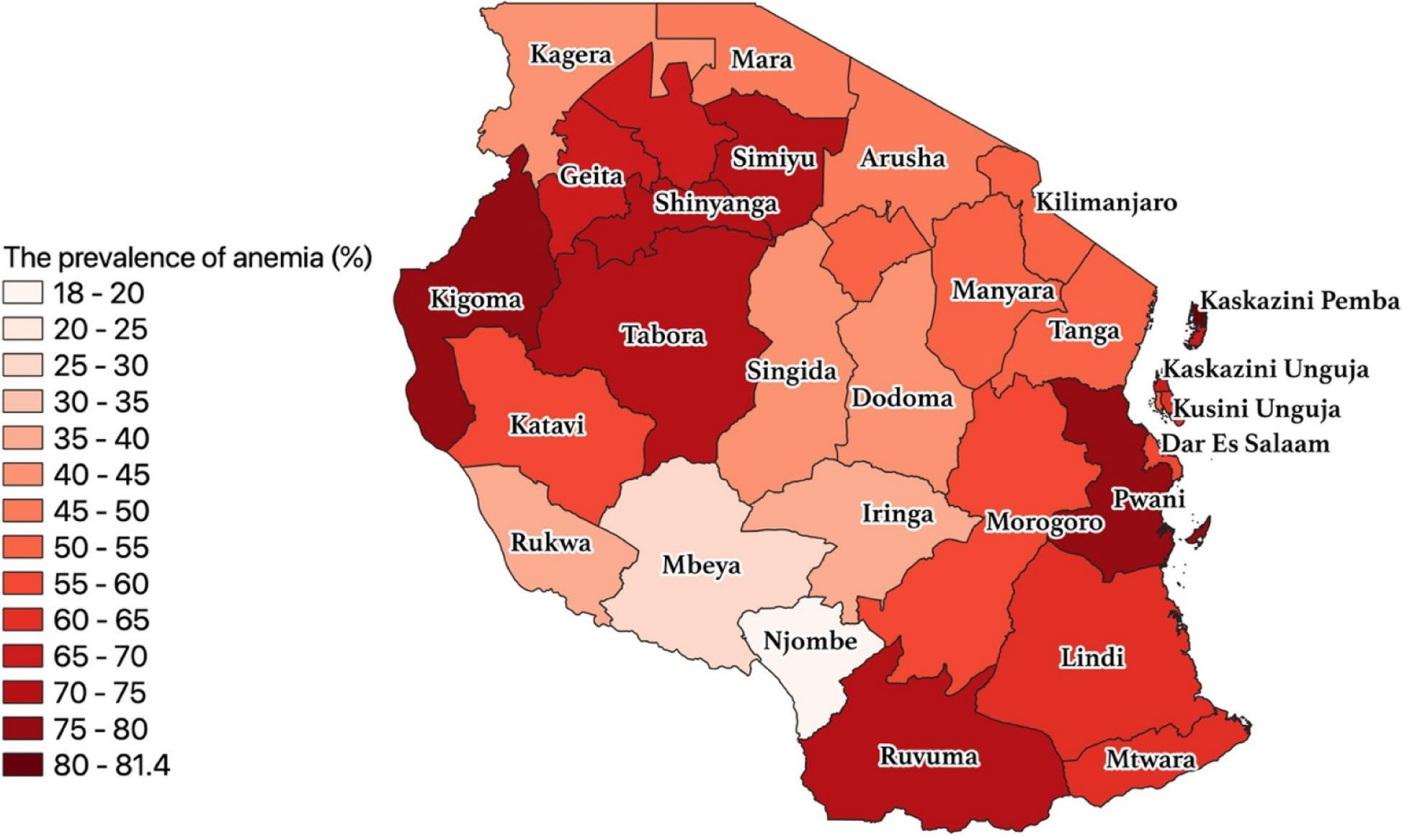
The GIS mapping of the regional burdens of anemia among pregnant in 2015–2016 survey

In the most recent survey (2015–2016), anemia among pregnant women was highest in Kaskazini Pemba (81.4%) followed by Pwani region (79.6%), Kigoma (77.1%), Shinyanga (74.5%). Anemia was the lowest in Njombe region (18%). Figure 2 shows the burden of anemia as of 2015–2016 national representative survey.

### Characteristics of anemia among pregnant women in Tanzania

Table 2 shows the descriptive characteristics of pregnant women in relation to anemia status between the two survey years. The burden of anemia among pregnant women was higher in two age extremes in both surveys. Younger women especially below 20 years old and older ones above 40 years old had higher burdens compared to other age categories. Although not statistically significant, education played a proactive role for anemia. Women with health insurance were more likely having lower burden of anemia. In both surveys, the larger the number of people in the household was related to a higher risk of anemia among pregnant women. Similar was for wealth index—lower risk of anemia among pregnant women living in wealthier households and households that were not food insecure.

**Table 2:** Characteristics of pregnant women in relation to the changing burden of anemia among pregnant women in Tanzania

**Variable**	**Anemia in 2004–2005**		**Anemia in 2015–2016**	
	**N**	**%**	**N**	**%**
**Individual and household characteristics**				
**Age (years)**				
15–19	126	65.1	150	63.9
20–24	158	54.3	153	54.8
25–29	169	62.7	149	55.3
30–34	77	50.6	87	52.3
35–39	57	52.7	66	55.7
40–49	38	64.6	33	67.8
*P*-Value		0.059		0.264
**Highest educational level**				
No education	174	60.7	126	63.4
Primary	429	57.4	411	56.2
Secondary education and above	23	55.2	102	53.9
*P*-Value		0.676		0.202
**Current marital status**				
Never married	54	70.6	59	59
Married	547	57.0	538	57.4
Others	25	63.2	42	51.9
*P*-value		0.087		0.751
**Use of Insecticide-treated bed nets**				
No bed net	407	55.7	239	54.6
Treated bed nets	115	65.2	37	49.6
Untreated bed nets	103	61.8	363	59.8
*P*-Value		0.145		0.215
**Has health insurance**^**a**^				
No			604	58.1
Yes			35	44.0
*P*-Value				0.040
**Type of residence**				
Urban	119	57.3	176	53.6
Rural	506	58.4	463	58.6
*P*-Value		0.828		0.187
**Number of household members**				
1–3	160	52.4	146	51.5
4–6	233	57.3	231	53.1
7–9	130	61.4	146	63.1
10 +	102	67.9	116	68.9
*P*-Value		0.032		0.005
**Wealth index**				
Poorest	139	59.4	153	57.9
Poorer	136	57.4	137	58.1
Middle	136	65.2	127	63.0
Richer	111	51.0	119	56.1
Richest	103	58.3	103	50.2
*P*-Value		0.281		0.285
**Food security**^**a**^				
Insecure			290	60.9
Secure			349	54.3
*P*-Value				0.077
**Low Feeding Frequency**^**a**^				
No			431	58.0
Yes			208	55.4
*P*-Value				0.429
**Maternal characteristics**				
**Number of children ever born**				
0	136	63.3	170	60.8
1	115	53.9	126	55.5
2	118	61.5	95	48.4
3	60	52.4	70	53.6
4 +	197	57.7	178	62.4
*P*-Value		0.342		0.088
**Age at the first childbirth (years)**				
0–19	338	57.7	310	58.0
20–49	152	55.3	158	52.1
*P*-Value		0.559		0.182
**Gestation age at ANC booking**				
0–3	36	54.5	74	57.4
4–6	341	57.6	283	57.5
7–9	45	58.1	33	57.5
*P*-Value		0.887		1.000
**Number of ANC visit**				
0–3	177	62.3	234	61.7
4 and above	254	54.2	167	53.4
*P*-Value		0.072		0.060
**Provision of IFA**				
No	178	60.7	98	61.2
Yes	253	55.0	304	57.1
*P*-Value		0.199		0.456
**Place of delivery**				
At home	250	58.8	188	63.5
Public health facility	154	56.9	179	54.8
Private health facility	31	50.3	28	46.6
*P*-Value		0.592		0.048
**Deworming**^**a**^				
No			161	61.5
Yes			240	56.0
*P*-Value				0.220
**Antimalaria given**				
No	292	57.6	149	63.0
Yes	138	56.9	251	55.4
*P*-Value		0.863		0.075

The *P*-values were taken from Pearson’s Chi-square test

^a^Data was available for only one dataset

With regards to reproductive and child characteristics, descriptive analyses suggest that although not significant, the burden of anemia was higher among women who gave birth to their first child at the age of 19 and below. Those with higher number of ANC attendances were less likely to have anemia. Anemia burden was low among those given iron tablets. In both surveys, the prevalence of anemia was higher among those who gave their previous birth at home compared to health facilities, those who were not dewormed, and not given antimalaria.

### Net change of anemia among pregnant women in Tanzania

After adjusting for individual, households, reproductive, and child characteristics that seems to have some association with anemia in the general characteristics above, this study found no significant change or decline of anemia among pregnant women in Tanzania between 2005 and 2015, AOR 0.964, 95% CI (0.774–1.202), *p* = 0.747 (Table 3). In this particular regression analyses, we adjusted for age, highest education level, current marital status, type of residence, number of household members, wealth index, and number of children ever born. In this particular analysis we also adjusted for survey weights.

**Table 3:** Anemia in pregnancy in TDHS 2015–2016 compared to TDHS 2004–2005

**Variable**	**AOR**	**95% CI**	***P-Value***
**Survey year**			
2004–2005	Ref		
2015–2016	0.964	0.774–1.202	0.747

OR adjusted for age, highest education level, current marital status, type of residence, number of household members, wealth index, and number of children ever born. Survey weights were adjusted

### The current burden of anemia among pregnant women and its determinants in Tanzania

After adjusting for covariates and confounders, the analysis based on TDHS 2015–2016 indicated that there was no difference in the prevalence of anemia among pregnant women between rural and urban areas in Tanzania (Table 4). Compared pregnant women living in households with up to 3 members, those in bigger size households were more likely to have anemia. For example, compared with households with up to three household members, pregnant women in households with up to six members were 1.3 times more likely to suffer from anemia (AOR = 1.285, 95% CI = 1.05–1.566, *p* = 0.013); in households with up to 9 people were more than 1.5 times more likely to suffer from anemia (AOR = 1.541, 95% CI = 1.241–1.913, *p* < 0.001) and those with more than ten members were more than twice more likely to suffer from anemia (AOR = 2.026, 95% CI = 1.603–2.560, *p* < 0.001). Women in higher than the first and subsequent wealth quintile were less likely to suffer from anemia, though such association did not reach a statistically significant level.

**Table 4:** Remaining factors associated with anemia in pregnancy, TDHS 2015–2016

**Variable**	**AOR**	**95%CI**	***P*-Value**
**Household characteristics**			
**Type of residence**			
Urban	1.000		
Rural	1.071	0.902—1.271	0.432
**Number of household members**			
1–3	1.000		
4–6	1.285**	1.054—1.566	0.013
7–9	1.541***	1.241—1.913	0.000
10 +	2.026***	1.603—2.560	0.000
**Wealth index**			
Poorest	1.000		
Poorer	1.080	0.899—1.298	0.410
Middle	1.045	0.868—1.259	0.640
Richer	1.122	0.922—1.367	0.250
Richest	1.198	0.931—1.542	0.160
**Individual Characteristics**			
**Age**			
15–19	1.000		
20–24	0.762**	0.592—0.979	0.034
25–29	0.678**	0.505—0.912	0.010
30–34	0.646***	0.464—0.901	0.010
35–39	0.799	0.561—1.138	0.214
40–49	0.764	0.527—1.108	0.156
**Highest educational level**			
No education	1.000		
Primary	0.691***	0.590—0.808	0.000
Secondary education and above	0.742***	0.597—0.923	0.007
**Current marital status**			
Never married	1.000		
Married	1.172	0.909—1.510	0.221
Other	1.124	0.839—1.505	0.434
**Number of children ever born**			
1	1.000		
2	0.942	0.769—1.155	0.567
3	0.778**	0.609—0.993	0.044
4 +	0.819	0.627—1.070	0.143
**Food Security**			
Insecure	1.000		
Secure	0.887*	0.785—1.001	0.052
**Low Feeding Frequency**			
No	1.000		
Yes	1.006	0.886—1.142	0.930
**Covered by health insurance**			
No	1.000		
Yes	0.701***	0.556—0.883	0.003
**Antimalaria given**			
No	1.000		
Yes	0.877**	0.775—0.994	0.040
**Place of delivery**			
At home	1.000		
Public health facility	0.840**	0.733—0.963	0.013
Private health facility	0.639***	0.520—0.786	0.000
**Number of ANC visit**			
0–3	1.000		
4 and above	0.889*	0.791—1.000	0.050
**Age at the first childbirth (years)**			
0–19	1.000		
20–49	1.032	0.900—1.184	0.652

Logistic regression with household-level random intercepts was used. Survey weights were also adjusted

^*^p < 0.1, ** *p* < 0.05, *** *p* < 0.01

Anemia was rampant among young pregnant women aged 15–19 years. Pregnant women aged between 20–34 years of age were less likely to succumb anemia compared to young pregnant women aged 15–19 years of age. For example, those between 20–24 were 24% less likely to suffer from anemia (*p* = 0.034), those between 25–29 were 32% less likely to have anemia (*p* = 0.010), and those between 30–34 were 35% less likely to suffer from anemia (*p* = 0.010), compared to late teenage pregnant mothers. Although older women were less likely to succumb anemia compared to teenage pregnant mothers, such association did not reach a statistically significant level.

Any level of education among pregnant women in Tanzania was beneficial and protective against anemia in pregnancy compared to no education at all. For example, compared to pregnant women with no education, those with primary education were 31% less likely to succumb from anemia (*p* < 0.001) and those with secondary school and above education were 26% less likely to succumb from anemia (*p* = 0.007). Pregnant women with previous birth experience were more likely not to have anemia compared to those whose pregnancy was the first. For example, those with three previous pregnancies were 22% less likely to suffer from anemia compared to those with the current pregnancy (*p* = 0.044).

Food insecurity has a role to play in anemia during pregnancy. Although the association did not reach a statistically significant level, compared to women in food insecure households, those from households with adequate food were 11% less likely to suffer from anemia (*p* = 0.052). Likewise, those who were covered by some form of health insurance were 30% less likely to succumb from anemia (*p* = 0.003). Women who were given antimalaria drugs during pregnancy were 12% less likely to suffer from anemia during pregnancy (*p* = 0.040). Place of delivery during the previous pregnancy seems to have a role in predicting the current pregnancy’s anemia. Those who delivered in public health facilities and private health facilities were 26% (*p* = 0.013) and 36% (*p* < 0.001) less likely to succumb from anemia in the current pregnancy compared to those who delivered at home. Women who attended ANC clinics in required frequency were 11% less likely to suffer from anemia in pregnancy (*p* = 0.050).

## Discussion

The burden of anemia did not decline among pregnant women in Tanzania between 2005 and 2015. After those 10 years, anemia was more prevalent in pregnant women (57%) compared with women of reproductive age (44%) in Tanzania mainland and Zanzibar. This unprecedented burden among pregnant women is attributed largely to moderate anemia contributing up to 30% of the total burden. In regional comparison, anemia burden has increased in 16 regions while 15 regions of Tanzania observed a modest to significant reduction. Such variation coupled with increment in other regions calls for regional specific and tailored interventions to address anemia. The current burden of anemia among pregnant women in Tanzania is associated with number of household members, woman’s age, education level, number of previous children, insurance coverage, use of antimalaria during pregnancy, place of delivery, and frequency of ANC attendance.

The prevalence of anemia in pregnancy in Tanzania is worrisome. In our previous publications, anemia among other subpopulations varied with regions, but there was a notable decline within the 10 years of the surveys [20]. In the current analysis, anemia has exhibited regional differences, however, a non-significant decline was observed through the 10 years between surveys. Pregnant women are more vulnerable to anemia because of the increased iron and folic acid deficiency owing to the growing fetus [26, 27]. The low coverage of the WHO recommended four or more ANC visits in Tanzania may reduce the probability of detecting and treating the potential anemia during pregnancy [13]. Besides, the low coverage of the WHO recommended at least three doses of SP during ANC visits in Tanzania may cause failure in preventing malaria, which is also a contributor to anemia [13]. Furthermore, the low adherence to iron-folic acid supplementation during ANC visits makes this treatment of iron and folic acid deficiency less effective [26]. Since anemia in pregnancy presents a common and potentially reversible risk factor associated with maternal mortality, this stagnation in decline may be a substantial hindrance to reducing maternal mortality [5, 6, 12, 13]. Addressing anemia among pregnant women is therefore an important entry point to attain SDGs.

Anemia in pregnancy may be caused by nutritional inadequacy, particularly on the lack of essential micronutrients such as iron and folic acid [15, 16]; diseases such as parasitic infestation, particularly hookworms [17]; and physiological challenges owing to the growing fetus and its nutritional demands. Owing to the high burden of anemia in the general population [13], majority of women would conceive with already challenged hemoglobin levels [14]. Pregnancy at a younger age is a risk factor to maternal challenges including anemia [28, 29]. Like in other contexts [30], the burden of anemia was higher among the youngest age band of pregnant women and those with demographic disadvantages including lack of or low education attainment. Apart from conception at the younger age, pregnancy at this tender age denies women opportunities to stay in school, accumulate knowledge, skills, and competencies to master life’s skills including having better nutrition. Moreover, at this age, they are succumbed to a vicious cycle of poverty and dependency owing low education level, subjecting them to many other demographic disadvantages. This study found association between anemia in pregnancy with age, low education level, and insurance coverage.

A number of interventions are in place to combat anemia in pregnancy and therefore maternal mortality. For example, early antenatal booking is highly recommended to enable expectant mothers are given supplements, preventive therapy with antimalaria, and delivery planning. This study found a clear association between anemia in pregnancy with uptake of antimalaria, frequency attendance to antenatal care, and place of delivery. Such findings are not different from other contexts [31, 32], and call for strengthening quality of maternal care, advocating for ANC attendance, and facility delivery.

These analyses showed a difference between regions in Tanzania like in other subpopulations [20]. Like in other contexts [15], this can be explained by factors such as food preferences and feeding restrictions that go hand in hand with differences in traditions and customs [22, 33]; food security [22]; agricultural activities, access to and coverage of essential preventive interventions including antenatal services, prevention and treatment of malaria, and iron and folic acid provision [26]. Women in most of food rich regions have lower magnitude of anemia. Iron is rich in sources of foods that adults consume in these areas where food is plenty and therefore in favor of their pre-pregnancy and pregnancy iron stores [14]. At least 15 regions showed a decline of anemia in the period of 10 years, however, 16 others had a worsened anemia burden in the same population. Such regions with increased burden including Kigoma Kigoma, Ruvuma, Tabora, and Lindi, need targeted interventions. This can be achieved through identifying local factors and therefore have impactful solutions.

Evidence from this study should be carefully interpreted owing to the following limitations: Firstly, we present secular trends over two cycles of DHS data, therefore the participants may not be the same in each survey. Secondly, this was a secondary data analysis in which we could only analyze the data that were present. Variables including ownership of health insurance, food security, low feeding frequency, and deworming in the last pregnancy are not available in the 2004–2005 dataset. These variables are important concerning anemia, and, therefore, the inability to compare them poses a limitation to current reporting. Lastly, since DHS surveys are cross-sectional, there is no attempt to identify causal relations in this study.

## Conclusions

Anemia in pregnancy has not declined over the past 10 years despite efforts to address maternal health challenges in Tanzania. Such unprecedented burden remains prevalent among 57% of pregnant women in the country. The regional variation of the burden of anemia in Tanzania calls for further identification of specific regional determinants and therefore designing tailored interventions. This study found important determinants of anemia including number of household members, woman’s age, education level, number of previous children, insurance coverage, use of antimalaria during pregnancy, place of delivery, and frequency of ANC attendance. Addressing such factors and further studies to address further local and regional determinants will help Tanzania attaining the speedy decline in the burden of anemia among pregnant women. For nutrition-specific interventions, it’s crucial to improve the iron and folic acid supplement coverage and to increase dietary diversity through food-based strategies early on in pregnancy, accompanied by social and behavior-change communication strategies. Of paramount importance, nutrition-sensitive interventions such as ensuring girls are well educated and stay in school beyond primary school education, health insurance coverage through social and community insurance schemes, strengthening maternal and child health services through early ANC booking and quality improvement, prevention of malaria and hookworm infestation, remain critical.

## Supplementary Information

**Additional file 1.**

## Data Availability

All datasets are available through request from the DHS website.

## Abbreviations

TDHS: Tanzania Demographic and Health Surveys
GIS: Geographic Information System
AOR: Adjusted Odds Ratio
ANC: Antenatal care
WRA: Women of reproductive age
Hb: Hemoglobin
NBS: National Bureau of Statistics
ZBS: Zanzibar Bureau of Statistics
USAID: U.S. Agency for International Development
MEASURE: Monitoring and Evaluation to Assess and Use Results
SDGs: Sustainable Development Goals

## References

[1] World Health organization (2015). The global prevalence of anaemia in 2011.

[2] Stevens GA, Finucane MM, De-Regil LM, Paciorek CJ, Flaxman SR, Branca F, Peña-Rosas JP, Bhutta ZA, Ezzati M, Nutrition Impact Model Study Group (Anaemia) (2013). Global, regional, and national trends in haemoglobin concentration and prevalence of total and severe anaemia in children and pregnant and non-pregnant women for 1995–2011: a systematic analysis of population-representative data. Lancet Glob Health.

[3] Kemppinen L, Mattila M, Ekholm E, Pallasmaa N, Törmä A, Varakas L, Mäkikallio K. Gestational iron deficiency anemia is associated with preterm birth, fetal growth restriction, and postpartum infections. J Perinat Med. 2020:jpm-2020–0379. 10.1515/jpm-2020-0379. Epub ahead of print. PMID: 33554586. 10.1515/jpm-2020-0379 33554586

[4] Scholl TO (2005). Iron status during pregnancy: setting the stage for mother and infant. Am J Clin Nutr.

[5] Brabin BJ, Hakimi M, Pelletier D (2001). An analysis of anemia and pregnancy-related maternal mortality. J Nutr.

[6] Smith C, Teng F, Branch E, Chu S, Joseph KS (2019). Maternal and perinatal morbidity and mortality associated with anemia in pregnancy. Obstet Gynecol.

[7] Chu FC, Shen-Wen Shao S, Lo LM, Hsieh TT, Hung TH (2020). Association between maternal anemia at admission for delivery and adverse perinatal outcomes. J Chin Med Assoc.

[8] Meazaw MW, Chojenta C, Muluneh MD, Loxton D (2020). Systematic and meta-analysis of factors associated with preeclampsia and eclampsia in sub-Saharan Africa. PLoS One.

[9] Yadav G, Chambial S, Agrawal N, Gothwal M, Kathuria P, Singh P, Sharma P, Sharma PP (2020). Blood lead levels in antenatal women and its association with iron deficiency anemia and adverse pregnancy outcomes. J Family Med Prim Care.

[10] Chang S, Zeng L, Brouwer ID, Kok FJ, Yan H (2013). Effect of iron deficiency anemia in pregnancy on child mental development in rural China. Pediatrics.

[11] Finkelstein JL, Kurpad AV, Bose B, Thomas T, Srinivasan K, Duggan C (2020). Anaemia and iron deficiency in pregnancy and adverse perinatal outcomes in Southern India. Eur J Clin Nutr.

[12] Bwana VM, Rumisha SF, Mremi IR, Lyimo EP, Mboera LEG (2019). Patterns and causes of hospital maternal mortality in Tanzania: A 10-year retrospective analysis. PLoS One.

[13] MoHCDGEC Ministry of Health CD, Gender, Elderly and Children - MoHCDGEC/Tanzania Mainland, MOH Ministry of Health - MoH/Zanzibar, NBS National Bureau of Statistics - NBS/Tanzania, OCGS Office of Chief Government Statistician - OCGS/Zanzibar, ICF. Tanzania demographic and health survey and malaria indicator survey 2015 - 2016. Dar es Salaam: MoHCDGEC, MoH, NBS, OCGS, and ICF; 2016.

[14] Ganatra BR, Hirve SS (1995). Unsafe motherhood: the determinants of maternal mortality. J Indian Med Assoc.

[15] Gibore NS, Ngowi AF, Munyogwa MJ, Ali MM (2020). Dietary habits associated with anemia in pregnant women attending antenatal care services. Curr Dev Nutr.

[16] Schmiegelow C, Msemo OA, Møller SL, Nielsen BB, Paulsen CB, Ødum L, Theander TG, Kavishe RA, Lusingu JPA, Minja DT, Bygbjerg IC (2019). Preconceptional factors associated with haemoglobin concentration in early pregnancy: a community-based cohort study in rural northeastern Tanzania. Trop Med Int Health.

[17] Kalinjuma AV, Darling AM, Mugusi FM, Abioye AI, Okumu FO, Aboud S, Masanja H, Hamer DH, Hertzmark E, Fawzi WW (2020). Factors associated with sub-microscopic placental malaria and its association with adverse pregnancy outcomes among HIV-negative women in Dar es Salaam, Tanzania: a cohort study. BMC Infect Dis.

[18] Shija AE, Rumisha SF, Oriyo NM, Kilima SP, Massaga JJ (2019). Effect of Moringa Oleifera leaf powder supplementation on reducing anemia in children below two years in Kisarawe District, Tanzania. Food Sci Nutr.

[19] Ngasala B, Matata F, Mwaiswelo R, Mmbando BP (2019). Anemia among schoolchildren with malaria and soil-transmitted helminth coinfections after repeated rounds of mass drug administration in Muheza district. Tanzania Am J Trop Med Hyg.

[20] Sunguya BF, Zhu S, Paulo LS, Ntoga B, Abdallah F, Assey V, Mpembeni R, Huang J (2020). Regional disparities in the decline of anemia and remaining challenges among children in Tanzania: analyses of the Tanzania demographic and health survey 2004–2015. Int J Environ Res Public Health.

[21] Project Locations. The World Bank, US Washington. 2021. https://maps.worldbank.org. Accessed 20 Feb 2021.

[22] Sunguya BF, Poudel KC, Mlunde LB, Urassa DP, Yasuoka J, Jimba M (2014). Poor nutrition status and associated feeding practices among HIV-positive children in a food secure region in Tanzania: a call for tailored nutrition training. PLoS One.

[23] NBS, Macro O. Tanzania demographic and health survey 2004–05. Dar es Salaam: NBS/Tanzania and ORC Macro; 2005.

[24] Croft TN, Marshall AMJ, Allen CK (2018). Guide to DHS statistics.

[25] Ministry of Health, Community Development, Gender, Elderly and Children - MoHCDGEC/Tanzania Mainland, Ministry of Health - MoH/Zanzibar, National Bureau of Statistics - NBS/Tanzania, Office of Chief Government Statistician - OCGS/Zanzibar, and ICF (2016). Tanzania demographic and health survey and malaria indicator survey 2015–2016.

[26] Lyoba WB, Mwakatoga JD, Festo C, Mrema J, Elisaria E (2020). Adherence to iron-folic acid supplementation and associated factors among pregnant women in Kasulu communities in north-western Tanzania. Int J Reprod Med.

[27] Etheredge AJ, Premji Z, Gunaratna NS, Abioye AI, Aboud S, Duggan C, Mongi R, Meloney L, Spiegelman D, Roberts D, Hamer DH, Fawzi WW (2015). Iron supplementation in iron-replete and nonanemic pregnant women in Tanzania: a randomized clinical trial. JAMA Pediatr.

[28] Karaçam Z, Kizilca Çakaloz D, Demir R. The impact of adolescent pregnancy on maternal and infant health in Turkey: systematic review and meta-analysis. J Gynecol Obstet Hum Reprod. 2021:102093. 10.1016/j.jogoh.2021.102093. Epub ahead of print. PMID: 33592347. 10.1016/j.jogoh.2021.102093 33592347

[29] Massawe SN, Ronquist G, Nyström L, Lindmark G (2002). Iron status and iron deficiency anaemia in adolescents in a Tanzanian suburban area. Gynecol Obstet Invest.

[30] Rahman MA, Rahman MS, Aziz Rahman M, Szymlek-Gay EA, Uddin R, Islam SMS (2021). Prevalence of and factors associated with anaemia in women of reproductive age in Bangladesh, Maldives and Nepal: Evidence from nationally-representative survey data. PLoS One.

[31] Nyamu GW, Kihara JH, Oyugi EO, Omballa V, El-Busaidy H, Jeza VT (2020). Prevalence and risk factors associated with asymptomatic Plasmodium falciparum infection and anemia among pregnant women at the first antenatal care visit: a hospital based cross-sectional study in Kwale County, Kenya. PLoS One.

[32] Rabiu OR, Dada-Adegbola H, Kosoko AM, Falade CO, Arinola OG, Odaibo AB, Ademowo OG (2020). Contributions of malaria, helminths, HIV and iron deficiency to anaemia in pregnant women attending ante-natal clinic in SouthWest Nigeria. Afr Health Sci.

[33] Darmawati D, Nizwan-Siregar T, Hajjul K, Tahlil T (2020). Exploring Indonesian mothers’ perspectives on anemia during pregnancy: a qualitative approach. Enferm Clin.

