# Supplementary material for: High burden of anemia among pregnant women in Tanzania: a call to address its determinants
Source: Nutr J. 2021 Jul 8;20:65. doi: 10.1186/s12937-021-00726-0 (PMC8268339; doi:10.1186/s12937-021-00726-0)
Supplement: Supplementary file 1 — Additional file 1. [file 12937_2021_726_MOESM1_ESM.docx]

**Supplementary Table 1**

| **Region** | **Prevalence 2005** | **Prevalence 2015** | **Percentage change** | **P-value** |
| --- | --- | --- | --- | --- |
| Dodoma | 55.6 | 43.2 | -12.4 | 0.318 |
| Arusha | 41.8 | 49 | 7.2 | 0.510 |
| Kilimanjaro | 49.4 | 50.2 | 0.8 | 0.965 |
| Tanga | 64.1 | 54 | -10.1 | 0.410 |
| Morogoro | 58.7 | 59.6 | 0.9 | 0.938 |
| Pwani | 85.9 | 79.7 | -6.2 | 0.587 |
| Dar Es Salam | 61.1 | 56.2 | -4.9 | 0.678 |
| Lindi | 50.6 | 62.2 | 11.6 | 0.500 |
| Mtwara | 57.4 | 61.8 | 4.4 | 0.737 |
| Ruvuma | 52.2 | 71.9 | 19.7 | 0.275 |
| Iringa | 34.5 | 37.1 | 2.6 | 0.867 |
| Mbeya | 35.3 | 26.6 | -8.7 | 0.413 |
| Singida | 59.7 | 45 | -14.7 | 0.079 |
| Tabora | 60.6 | 71 | 10.4 | 0.242 |
| Rukwa | 47.7 | 35.8 | -11.9 | 0.289 |
| Kigoma | 53 | 77.1 | 24.1 | 0.004 |
| Shinyanga | 71.9 | 74.5 | 2.6 | 0.789 |
| Kagera | 39 | 43.3 | 4.3 | 0.639 |
| Mwanza | 77.5 | 68.3 | -9.2 | 0.226 |
| Mara | 72.9 | 49.7 | -23.2 | 0.006 |
| Manyara | 60 | 51.2 | -8.8 | 0.451 |
| Njombe | 34.5 | 18 | -16.5 | 0.229 |
| Katavi | 47.7 | 55.6 | 7.9 | 0.698 |
| Simiyu | 71.9 | 70.4 | -1.5 | 0.886 |
| Geita | 77.5 | 69.3 | -8.2 | 0.312 |
| Kaskazini Unguja | 79.1 | 65.1 | -14 | 0.175 |
| Kusini Unguja | 48 | 60.3 | 12.3 | 0.356 |
| Mjini Magharibi | 66.9 | 53.7 | -13.2 | 0.105 |
| Kaskazini Pemba | 68.9 | 81.4 | 12.5 | 0.213 |
| Kusini Pemba | 58.1 | 69 | 10.9 | 0.195 |
